# Lung cancer incidence and survival in different ethnic groups in South East England

**DOI:** 10.1038/bjc.2011.282

**Published:** 2011-08-23

**Authors:** R H Jack, E A Davies, H Møller

**Affiliations:** 1 King's College London, Thames Cancer Registry, 1st Floor Capital House, 42 Weston Street, London SE1 3QD, UK

**Keywords:** ethnicity, lung cancer, incidence, survival

## Abstract

**Background:**

This study aimed to examine the incidence and survival of lung cancer patients from several different ethnic groups in a large ethnically diverse population in the United Kingdom.

**Methods:**

Data on residents of South East England diagnosed with lung cancer between 1998 and 2003 were extracted from the Thames Cancer Registry database. Age- and socioeconomic deprivation-standardised incidence rate ratios were calculated for males and females in each ethnic group. Overall survival was examined using Cox regression, adjusted for age, socioeconomic deprivation, stage of disease and treatment. Results are presented for White, Indian, Pakistani, Bangladeshi, Black Caribbean, Black African and Chinese patients, apart from female survival results where only the White, South Asian and Black ethnic groups were analysed.

**Results:**

Compared with other ethnic groups of the same sex, Bangladeshi men, White men and White women had the highest incidence rates. Bangladeshi men had consistently higher survival estimates compared with White men (fully adjusted hazard ratio 0.46; *P*<0.001). Indian (0.84; *P*=0.048), Black Caribbean (0.87; *P*=0.47) and Black African (0.68; *P*=0.007) men also had higher survival estimates. South Asian (0.73; *P*=0.006) and Black (0.74; *P*=0.004) women had higher survival than White women.

**Conclusion:**

Smoking prevention messages need to be targeted for different ethnic groups to ensure no groups are excluded. The apparent better survival of South Asian and Black patients is surprising, and more detailed follow-up studies are needed to verify these results.

Lung cancer is the most common cancer worldwide, accounting for 13% of all incident cancers (excluding skin cancer) in 2008 (Ferlay *et al*, 2010). The age-standardised lung cancer incidence rates, calculated using the world standard population, were highest in North America, Eastern Asia and across Europe for males, and in North America, Northern Europe, Eastern Asia, Australia and New Zealand for females (Ferlay *et al*, 2010).

In the United Kingdom, studies have shown lower lung cancer incidence rates in males and females with South Asian names (those of Indian, Pakistani or Bangladeshi origin) compared with their counterparts with non-South Asian names (Winter *et al*, 1999; Day *et al*, 2010). Recent work has used the 2001 Census ethnic groups, either at a broad level or using the more detailed subgroups. In Leicester, the more specific Indian male and female groups have been shown to have lower lung cancer rates than the corresponding White British groups (Ali *et al*, 2010). A recent report on cancer incidence and survival in England found that White men and women had higher incidence rates than South Asian, Black, Chinese and mixed ethnic groups of the same sex (National Cancer Intelligence Network, 2009). Using country of birth as a proxy for ethnicity, statistically significantly lower standardised incidence ratios (adjusted for age and year of diagnosis) were found in men born in South Asia and in the West Indies, compared with all men (Harding and Rosato, 1999). The very small number of lung cancer cases diagnosed in women born in South Asia and the West Indies meant low standardised incidence ratios, with very wide confidence intervals, were generated in this study.

Analyses of lung cancer survival have been more common in the United States, and have tended to find no difference between Black and White patients when treatment received is taken into account (Greenwald *et al*, 1998; Bach *et al*, 1999; Mulligan *et al*, 2006). However, Goggins and Wong (2009) found Indian/Pakistani and Chinese patients had better overall survival compared with White patients, adjusted for demographic and disease variables including age and stage. In England, higher 1- and 3-year relative survival was found in Asian lung cancer patients compared with White and Black groups of the same sex (National Cancer Intelligence Network, 2009).

## Materials and methods

In the United Kingdom, cancer registries record the occurrence of cancer in their resident populations. In the area covered by the Thames Cancer Registry (TCR), registration is initiated by clinical and pathological information received from hospitals and by information about deaths provided by the National Health Service Central Register through the Office for National Statistics. Trained cancer registration officers then extract further information on demographic details, disease stage and treatment in the first 6 months after diagnosis from individual medical records. The Thames Cancer Registry uses a simple four-level staging system, using information in the medical notes. This allows solid tumours to be assigned to categories based on whether the disease is local, has direct extension beyond the organ of origin, has regional lymph node involvement or has metastasised. Data are quality assured as they are added to the central database. Data on all male and female patients with lung (including trachea and bronchus) cancer (ICD-10 codes C33 and C34) diagnosed between 1998 and 2003 were extracted from the TCR database.

The Hospital Episode Statistics (HES) data set contains information collected from hospital Patient Administration Systems including self-assigned ethnicity, and is collated by the NHS Information Centre. All inpatient episodes for patients residing in South East England with cancer (or suspected cancer) admitted to NHS hospitals between April 1997 and March 2004 were obtained.

Ethnicity information from the HES data was matched with the TCR records using NHS number or sex, date of birth and postcode of residence. If there was no match from the HES data, ethnicity routinely recorded by TCR was used, where available. The groups used in the incidence analyses were White, Indian, Pakistani, Bangladeshi, Black Caribbean, Black African and Chinese. For the survival analyses, there were large enough numbers of male patients to analyse all of the above ethnic groups. However, the smaller number of female lung cancer cases meant that the broader White, South Asian (Indian, Pakistani, Bangladeshi and Asian Other) and Black (Black Caribbean, Black African and Black Other) groups were analysed for females.

Population data from the 2001 Census were available for local authorities in South East England. The Indices of Deprivation 2000 (Department of the Environment, Transport and the Regions, 2000) scores were also calculated for local authorities. Quintiles of the income domain were used to measure socioeconomic deprivation, creating population data stratified by age group, sex, ethnic group and five socioeconomic deprivation groups. Poisson regression analyses of incidence, adjusting for age and socioeconomic deprivation, were calculated separately for males and females by ethnic group. Overall survival was examined using Cox regression, with patients followed up until 31 December 2006. Results were sequentially adjusted for age, socioeconomic deprivation, stage of disease and treatment received.

## Results

There were 46 402 patients diagnosed with lung cancer, resident in South East England between 1998 and 2003: 28 145 males and 18 257 females. The number and proportion of patients in each ethnic group for males and females are shown in Table 1. There was a very small difference in the proportion of female (35.8%) and male (33.7%) lung cancer patients with no ethnicity information. The majority of male and female patients were coded as White. The male to female ratio varied between ethnic groups (Table 1). Although the ratio of males to females was 1.5 : 1 overall and 1.6 : 1 in the White ethnic group, the ratio was largest (8.8 : 1) in Bangladeshi patients.

After excluding 4 163 (9%) cases registered by death certificate information only, 28 803 (68%) patients had a known ethnicity. Survival analyses results are shown for 16 501 (64.2%) White, 142 (0.6%) Indian, 43 (0.2%) Pakistani, 95 (0.4%) Bangladeshi, 235 (0.9%) Black Caribbean, 58 (0.2%) Black African and 55 (0.2%) Chinese male patients with lung cancer. Results for 10 608 (64.1%) White, 96 (0.6%) South Asian and 107 (0.6%) Black female patients with lung cancer are also presented.

### Incidence

**Figure 1:**
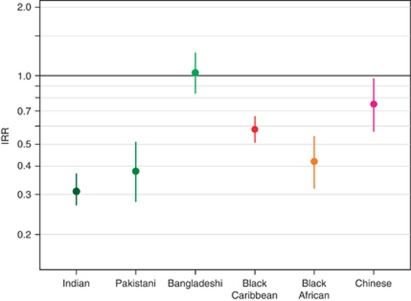
Poisson regression IRR and 95% CIs for male lung cancer diagnosed 1998–2003, South East England. Adjusted for age and socioeconomic deprivation, White men were used as the baseline group.

The age- and socioeconomic deprivation-standardised incidence rate ratios (IRRs) for male lung cancer patients are shown in Figure 1. Indian (0.31; 95% confidence interval (CI), 0.27–0.37), Pakistani (0.38; 95% CI 0.28–0.51), Black Caribbean (0.57; 95% CI 0.50–0.65) and Black African (0.42; 95% CI 0.32–0.54) men had much lower rate ratios compared with White men. Chinese men had a higher IRR (0.75; 95% CI 0.57–0.98) than other ethnic groups, and were more similar to the baseline group of White men. There was no statistically significant difference between Bangladeshi and White men's incidence rates (1.03; 95% CI 0.85–1.26).

**Figure 2:**
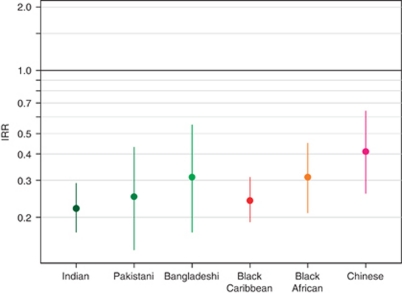
Poisson regression IRR and 95% CIs for female lung cancer 1998–2003, South East England. Adjusted for age and socioeconomic deprivation; White women were used as the baseline group.

In females, White women had the highest rate of lung cancer (Figure 2). Compared with White women, the highest IRR was for Chinese women and was only 0.41 (95% CI 0.26–0.64). All other ethnic groups’ IRRs were between 0.22 (Pakistani women) and 0.31 (Bangladeshi and Black African women). The differences between the White baseline and other ethnic groups were larger for women than for men.

### Survival

Results from the overall survival analysis in male lung cancer patients, sequentially adjusted for age, socioeconomic deprivation, stage of disease and treatment, are shown in Table 2. Bangladeshi men had the highest overall survival estimate, and this was largely unaffected by further adjustment (fully adjusted hazard ratio 0.46; *P*<0.001). In the fully adjusted model, Indian (0.84; *P*=0.048), Black Caribbean (0.87; *P*=0.047) and Black African (0.68; *P*=0.007) men also had statistically significant lower hazard ratios compared with the baseline of White men.

Table 3 shows the overall survival analysis results for female lung cancer patients, again adjusted for age, socioeconomic deprivation, stage and treatment. South Asian women consistently had better overall survival (0.73; *P*=0.006) and in the fully adjusted model, Black women had a similarly low hazard ratio (0.74; *P*=0.004).

For both male and female lung cancer patients, those living in the most affluent areas had better survival, although this was more evident for males (trend *P*<0.001) than for females (trend *P*=0.051). Patients who had a record of cancer surgery, radiotherapy or chemotherapy had better survival, for both males and females.

## Discussion

White and Bangladeshi men had the highest age- and socioeconomic deprivation-standardised incidence rates of lung cancer compared with other ethnic groups analysed. For women, the age-standardised incidence rate was much higher in the White group than in all other ethnic groups. South Asian and Black women had better survival estimates than White women. Bangladeshi men consistently had the best survival estimates, regardless of adjustments made.

Previous work has shown that Black, South Asian and Chinese men and women had lower incidence rates than their White counterparts in England (Harding and Rosato, 1999; Winter *et al*, 1999; National Cancer Intelligence Network, 2009; Ali *et al*, 2010; Day *et al*, 2010). The present study has confirmed that in South East England Indian, Pakistani, Black Caribbean, Black African and Chinese men and women have lower age- and socioeconomic deprivation-standardised incidence rates than White groups of the same sex. Previous studies carried out in the United Kingdom that used lung cancer mortality as an index for incidence, found that men born in Bangladesh had incidence rates which were similar to those of the general population (Wild *et al*, 2006) or men born in England and Wales (Harding *et al*, 2009). The results for South East England presented here found a similar pattern by examining actual incidence, and had a further methodological advantage of taking socioeconomic deprivation into account.

Several studies carried out in the United States have shown that Black and White lung cancer patients who receive the same treatment have similar survival (Greenwald *et al*, 1998; Bach *et al*, 1999; Mulligan *et al*, 2006). Asian-born lung cancer patients were found to have lower crude survival than US-born patients (Finlay *et al*, 2002), and Goggins and Wong (2009) found higher survival in Indian/Pakistani and Chinese patients after adjusting for demographic and disease variables, but not for treatment. In England, South Asian men and women had higher 1- and 3-year relative survival estimates than White and Black groups (National Cancer Intelligence Network, 2009). In the present study, Bangladeshi men, Black African men and South Asian women had higher survival than their White counterparts, after adjusting for age only. Besides taking socioeconomic deprivation, stage of disease and treatment received into account, Indian, Bangladeshi, Black Caribbean and Black African men, and South Asian and Black women had higher survival than White lung cancer patients.

This study includes a large, ethnically diverse population, and has therefore been able to examine incidence and survival for several ethnic groups with minimal need to use aggregated groups. However, some ethnic groups only had small numbers of lung cancer patients. It is important to make sure that good care is taken of all patients, and that this is not impeded by presenting as a rare event. The ability of these analyses to take both age and socioeconomic deprivation into account in examining the patterns of lung cancer incidence is an important step.

The much higher survival in Bangladeshi men was consistently found in all analyses and is therefore of particular interest. It is possible that this is because of a lack of accurate follow-up of these patients. The death information routinely used by the TCR is from death certificates that have been recorded and issued by the Office for National Statistics. A separate category of ‘informal deaths’ also exists. This is where a patient has been recorded on the NHS Care Records Service as having died, but no formal death certificate has been issued. To explore whether these informal deaths would explain the variation in survival, all male lung cancer patients who were recorded as still alive at the censor date of 31 December 2006 were matched with the Care Records Service, and updated death information on or before this date was extracted. The largest proportion of patients with updated death information was in the Bangladeshi group (13 out of 27, 48%). Small numbers of Indian (1 out of 17, 6%), Pakistani (1 out of 7, 14%), Black Caribbean (2 out of 23, 9%), Black African (2 out of 9, 22%) and Chinese (1 out of 4, 25%) patients were originally believed to be alive and had updated death information. A large number of White patients had new death information traced, but the proportion was low (103 out of 925, 11%). Incorporating this new information into the fully adjusted survival analysis had little effect on the hazard ratios for most ethnic groups. The Bangladeshi group result was attenuated (from 0.46; 95% CI 0.36–0.59 to 0.60; 95% CI 0.48–0.75), but was still statistically significantly higher than the White group estimate.

The histological lung cancer subtypes were also considered to attempt to understand the differences found in survival. After excluding patients registered from death certificate information only, the proportions of each histological subtype were calculated for males and females in each ethnic group. Higher proportions of better prognosis subtypes (such as adenocarcinoma and bronchioloalveolar carcinoma), and lower proportions of poorer prognosis subtypes (e.g., small cell) were found in South Asian and Black women compared with White women. Although Bangladeshi men had the highest survival estimate, they also had the largest proportion of small cell carcinomas of all the male ethnic groups. Adjusting for histological subtypes did not materially affect the survival results for males or females (data not shown).

There was variation in the recorded treatment in different ethnic groups. Although 14% of Bangladeshi men received cancer surgery within 6 months of diagnosis, only 8% of White men did, after adjusting for age, socioeconomic deprivation and stage of disease. There was little variation in the proportions of patients receiving radiotherapy, with the exception of a statistically significantly lower proportion of Bangladeshi men having radiotherapy compared with White men (21% *vs* 37%, *P*=0.002). Chemotherapy ranged from 15% in Pakistani men to 27% in Chinese men. The proportions of female lung cancer patients recorded as receiving different treatments were similar in the broad ethnic groups examined (data not shown). Adjusting for treatment made little difference to the survival analysis results (Tables 2 and 3).

The variation in the survival analyses was surprising. The possibility of patients leaving their country of residence to return to their country of birth and being lost to follow-up has been explored in other geographical areas. Raymond *et al* (1996) found better male lung cancer survival in patients who had been resident in Geneva for less time, and worse survival in all Swiss cancer patients compared with non-Swiss patients. Two mechanisms were put forward to explain this. First, the ‘healthy immigrant’ effect, where only those patients with good health are both motivated to migrate to another country and allowed in. Second, the ‘unhealthy emigrant’ effect, where it has been suggested that immigrants leave a country when they are diagnosed with a serious illness. Routinely used formal death certification information does not capture all deaths, and the extent of this appears to vary by ethnic group. However, this additional death information did not fully explain the apparent better survival in male Bangladeshi lung cancer patients. It is likely that some patients’ deaths are not recorded as formal or informal deaths, and remain lost to follow-up.

As lung cancer is closely linked to smoking, the patterns of incidence will reflect smoking habits in different ethnic groups. Although smoking status information was not available for the individuals in the present study, the second Health Education Authority survey examined smoking status in different ethnic groups around the same period (Johnson *et al*, 2000). Proportions of current or ex-regular smokers in different ethnic groups in England show that Bangladeshi men have a smoking prevalence very similar to the male population in England (58% and 60%, respectively), whereas the proportions were lower for African-Caribbean (48%), Indian (27%) and Pakistani men (37%) (Johnson *et al*, 2000). Smoking prevalence was lower in women than their male counterparts. For women, the proportion of current or ex-regular smokers was highest in the overall England population (48%), followed by African-Caribbean women (28%), and there were very low levels in Indian (4%), Pakistani (4%) and Bangladeshi (8%) women. If smoking increases, consequently lung cancer rates will also increase. The health messages of smoking prevention and cessation, therefore, needs to be targeted at everyone, with particular attention paid to groups with higher smoking rates. Issues such as language and relevancy of any health literature need to be considered to ensure no groups are excluded. For example, Bangladeshi men in Tower Hamlets perceived that nicotine replacement therapy products were aimed at White middle-class people (Croucher and Choudhury, 2007).

Future studies with more complete information on ethnicity and aspects of cancer care would be useful in clarifying equality in access to care and survival in different ethnic groups. Studies with more detailed follow-up, particularly paying attention to emigration out of the country, would lead to evidence about whether survival estimates in patients from ethnic groups in the United Kingdom are subject to bias.

## Figures and Tables

**Table 1:** Number, percentage and mean age in years of lung cancer patients diagnosed 1998–2003, South East England by sex and ethnic group, and male/female (M/F) ratio by ethnic group

	**Males**			**Females**			
	***n***	**%**	**Mean age (in years)**	***n***	**%**	**Mean age (in years)**	**M/F ratio**
White	17 412	61.9	71	11 211	61.4	71	1.6
Indian	153	0.5	68	61	0.3	65	2.5
Pakistani	44	0.2	65	12	0.1	57	3.7
Bangladeshi	97	0.3	66	11	0.1	56	8.8
Black Caribbean	240	0.9	66	62	0.3	64	3.9
Black African	59	0.2	62	28	0.2	60	2.1
Chinese	55	0.2	64	19	0.1	58	2.9
Other groups	595	2.1	67	323	1.8	68	1.8
Not known	9490	33.7	72	6530	35.8	74	1.5
Total	28 145	100.0	71	18 257	100.0	72	1.5

**Table 2:** Hazard ratios (HRs) and 95% confidence intervals (95% CIs) for overall survival in male patients diagnosed with lung cancer 1998–2003, South East England. Adjusted sequentially for age, socioeconomic deprivation, stage of disease and treatment

	**Adjusted for age**			**Adjusted for socioeconomic deprivation**			**Adjusted for stage**			**Adjusted for treatment**		
	**HR**	**(95% CI)**	***P*-value**	**HR**	**(95% CI)**	***P*-value**	**HR**	**(95% CI)**	***P*-value**	**HR**	**(95% CI)**	***P*-value**
*Ethnic group*												
White	1.00			1.00			1.00			1.00		
Indian	0.87	(0.73, 1.04)	*0.131*	0.86	(0.53, 1.05)	*0.091*	0.84	(0.70, 1.00)	*0.047*	0.84	(0.70, 1.00)	*0.048*
Pakistani	0.95	(0.69, 1.32)	*0.774*	0.92	(0.66, 1.28)	*0.627*	0.94	(0.68, 1.30)	*0.707*	0.94	(0.68, 1.31)	*0.721*
Bangladeshi	0.54	(0.42, 0.68)	<*0.001*	0.51	(0.40, 0.65)	<*0.001*	0.49	(0.39, 0.62)	<*0.001*	0.46	(0.36, 0.59)	<*0.001*
Black Caribbean	0.97	(0.85, 1.11)	*0.641*	0.93	(0.81, 1.07)	*0.306*	0.92	(0.80, 1.05)	*0.215*	0.87	(0.76, 1.00)	*0.047*
Black African	0.77	(0.58, 1.01)	*0.062*	0.74	(0.56, 0.98)	*0.037*	0.69	(0.52, 0.92)	*0.010*	0.68	(0.51, 0.90)	*0.007*
Chinese	0.91	(0.69, 1.20)	*0.498*	0.89	(0.68, 1.18)	*0.429*	0.84	(0.63, 1.10)	*0.199*	0.81	(0.62, 1.07)	*0.141*
Test for heterogeneity:												
*χ*^2^-test (6 d.f.)	32.0		*P*<0.0001	38.3		*P*<0.0001	46.7		*P*<0.0001	56.2		*P*<0.0001
												
*Socioeconomic deprivation*												
1 (Most affluent)				1.00			1.00			1.00		
2				1.05	(1.01, 1.10)	*0.024*	1.04	(1.00, 1.09)	*0.055*	1.04	(1.00, 1.09)	*0.067*
3				1.08	(1.03, 1.13)	*0.001*	1.08	(1.03, 1.13)	*0.001*	1.06	(1.02, 1.11)	*0.005*
4				1.11	(1.06, 1.15)	<*0.001*	1.10	(1.05, 1.14)	<*0.001*	1.07	(1.02, 1.11)	*0.003*
5 (Most deprived)				1.15	(1.10, 1.20)	<*0.001*	1.12	(1.07, 1.16)	<*0.001*	1.10	(1.05, 1.14)	<*0.001*
Test for trend:												
*χ*^2^-test (1 d.f.)				47.5		*P*<0.0001	31.2		*P*<0.0001	18.1		*P*<0.0001
												
*Treatment*												
Cancer surgery										0.24	(0.22, 0.26)	<*0.001*
Radiotherapy										0.68	(0.67, 0.70)	<*0.001*
Chemotherapy										0.65	(0.63, 0.68)	<*0.001*

Abbreviation: d.f.=degree of freedom.

The italicised figures are the *P*-values associated with the hazard ratios.

**Table 3:** Hazard ratios (HRs) and 95% confidence intervals (95% CIs) for overall survival in female patients diagnosed with lung cancer 1998–2003, South East England. Adjusted sequentially for age, socioeconomic deprivation, stage of disease and treatment

	**Adjusted for age**			**Adjusted for socioeconomic deprivation**			**Adjusted for stage**			**Adjusted for treatment**		
	**HR**	**(95% CI)**	***P*-value**	**HR**	**(95% CI)**	***P*-value**	**HR**	**(95% CI)**	***P*-value**	**HR**	**(95% CI)**	***P-*value**
*Ethnic group*												
White	1.00			1.00			1.00			1.00		
South Asian	0.76	(0.61, 0.95)	*0.017*	0.76	(0.15, 0.67)	*0.017*	0.78	(0.63, 0.97)	*0.029*	0.73	(0.59, 0.91)	*0.006*
Black	0.89	(0.73, 1.10)	*0.278*	0.87	(0.71, 1.08)	*0.204*	0.82	(0.67, 1.01)	*0.056*	0.74	(0.60, 0.91)	*0.004*
Test for heterogeneity:												
*χ*^2^-test (2 d.f.)	6.8		*P*=0.0333	7.3		*P*=0.0265	8.3		*P*=0.0155	15.8		*P*=0.0004
												
*Socioeconomic deprivation group*												
1 (Most affluent)				1.00			1.00			1.00		
2				1.05	(1.00, 1.11)	*0.068*	1.03	(0.98, 1.09)	*0.261*	1.01	(0.95, 1.07)	*0.771*
3				1.10	(1.04, 1.16)	<*0.001*	1.08	(1.02, 1.14)	*0.005*	1.06	(1.00, 1.12)	*0.042*
4				1.13	(1.08, 1.20)	<*0.001*	1.10	(1.05, 1.16)	<*0.001*	1.06	(1.00, 1.12)	*0.034*
5 (Most deprived)				1.11	(1.06, 1.17)	<*0.001*	1.06	(1.01, 1.12)	*0.021*	1.04	(0.99, 1.10)	*0.142*
Test for trend:												
*χ*^2^-test (1 d.f.)				21.2		*P*<0.0001	8.3		*P*=0.0039	3.8		*P*=0.0506
												
*Treatment*												
Cancer surgery										0.22	(0.21, 0.24)	<*0.001*
Radiotherapy										0.73	(0.71, 0.76)	<*0.001*
Chemotherapy										0.68	(0.65, 0.71)	<*0.001*

Abbreviation: d.f.=degree of freedom.

The italicised figures are the *P*-values associated with the hazard ratios.

## References

[bib1] Ali R, Barnes I, Kan SW, Beral V (2010) Cancer incidence in British Indians and British whites in Leicester, 2001–2006. Br J Cancer 103(1): 143–148 20551955 10.1038/sj.bjc.6605744 PMC2905295

[bib2] Bach PB, Cramer LD, Warren JL, Begg CB (1999) Racial differences in the treatment of early-stage lung cancer. N Engl J Med 341(16): 1198–1205 10519898 10.1056/NEJM199910143411606

[bib3] Croucher R, Choudhury SR (2007) Tobacco control policy initiatives and UK resident Bangladeshi male smokers: community-based, qualitative study. Ethn Health 12(4): 321–337 17701760 10.1080/13557850701300731

[bib4] Day M, Poole J, Bennett JA, Peake MD (2010) Changes in lung cancer incidence in South Asians in Leicester, 1990–2005. J Pub Health 32(2): 230–235 10.1093/pubmed/fdp090 19828680

[bib5] Department of the Environment, Transport and the Regions (2000) Indices of Deprivation 2000: Regeneration Research Summary No. 31. Stationery Office: London

[bib6] Ferlay J, Shin HR, Bray F, Forman D, Mathers C, Parkin DM (2010) Estimates of worldwide burden of cancer in 2008: GLOBOCAN 2008. Int J Cancer 127(12): 2893–2917 21351269 10.1002/ijc.25516

[bib7] Finlay GA, Joseph B, Rodrigues CR, Griffith J, White AC (2002) Advanced presentation of lung cancer in Asian immigrants: a case-control study. Chest 122(6): 1938–1943 12475830 10.1378/chest.122.6.1938

[bib8] Goggins WB, Wong G (2009) Cancer among Asian Indians/Pakistanis living in the United States: low incidence and generally above average survival. Cancer Causes Control 20(5): 635–643 19067192 10.1007/s10552-008-9275-x

[bib9] Greenwald HP, Polissar NL, Borgatta EF, McCorkle R, Goodman G (1998) Social factors, treatment, and survival in early-stage non-small cell lung cancer. Am J Public Health 88(11): 1681–1684 9807536 10.2105/ajph.88.11.1681 PMC1508564

[bib10] Harding S, Rosato M (1999) Cancer incidence among first generation Scottish, Irish, West Indian and South Asian migrants living in England and Wales. Ethn Health 4(1–2): 83–92 10887464 10.1080/13557859998218

[bib11] Harding S, Rosato M, Teyhan A (2009) Trends in cancer mortality among migrants in England and Wales, 1979–2003. Eur J Cancer 45(12): 2168–2179 19349162 10.1016/j.ejca.2009.02.029

[bib12] Johnson MRD, Owen D, Blackburn C (2000) Black and Minority Ethnic Groups in England: Second Health and Lifestyle Survey. Health Development Agency: London

[bib13] Mulligan CR, Meram AD, Proctor CD, Wu H, Zhu K, Marrogi AJ (2006) Unlimited access to care: effect on racial disparity and prognostic factors in lung cancer. Cancer Epidemiol Biomarkers Prev 15(1): 25–31 16434582 10.1158/1055-9965.EPI-05-0537

[bib14] National Cancer Intelligence Network (2009) Cancer Incidence and Survival by Major Ethnic Group in England, 2002–2006. http://library.ncin.org.uk/docs/090625-NCIN-Incidence_and_Survival_by_Ethnic_Group-Report.pdf, (Last accessed: 22 February 2011)

[bib15] Raymond L, Fischer B, Fioretta G, Bouchardy C (1996) Migration bias in cancer survival rates. J Epidemiol Biostat 1(3): 167–173

[bib16] Wild SH, Fischbacher CM, Brock A, Griffiths C, Bhopal R (2006) Mortality from all cancers and lung, colorectal, breast and prostate cancer by country of birth in England and Wales, 2001–2003. Br J Cancer 94(7): 1079–1085 16523198 10.1038/sj.bjc.6603031 PMC2361230

[bib17] Winter H, Cheng KK, Cummins C, Maric R, Silcocks P, Varghese C (1999) Cancer incidence in the south Asian population of England (1990–92). Br J Cancer 79(3–4): 645–654 10027344 10.1038/sj.bjc.6690102 PMC2362427

